# Supplementary material for: Mechanisms governing the pioneering and redistribution capabilities of the non-classical pioneer PU.1
Source: Nat Commun. 2020 Jan 21;11:402. doi: 10.1038/s41467-019-13960-2 (PMC6972792; doi:10.1038/s41467-019-13960-2)
Supplement: Supplementary file 7 — Source data [file 41467_2019_13960_MOESM7_ESM.zip › Source_Data/Figure5/Figure5A_MotifScanOutput/homerResults/motif24.similar.html]

motif24

## Information for motif24

C
G
T
A
A
C
T
G
T
A
C
G
G
T
A
C
C
G
T
A
A
T
C
G
A
T
C
G
A
G
T
C
A
G
T
C
A
C
G
T
A
T
G
C
A
G
T
C
A
G
C
T
C
G
A
T
  
Reverse Opposite:  

C
G
T
A
C
T
G
A
C
T
A
G
T
A
C
G
C
G
T
A
A
C
T
G
A
C
T
G
A
T
G
C
A
T
G
C
A
C
G
T
A
C
T
G
A
T
G
C
A
G
T
C
A
C
G
T
  

|  |  |
| --- | --- |
| p-value: | 1e-44 |
| log p-value: | -1.025e+02 |
| Information Content per bp: | 1.947 |
| Number of Target Sequences with motif | 65.0 |
| Percentage of Target Sequences with motif | 2.16% |
| Number of Background Sequences with motif | 58.0 |
| Percentage of Background Sequences with motif | 0.13% |
| Average Position of motif in Targets | 147.1 +/- 90.3bp |
| Average Position of motif in Background | 239.2 +/- 157.1bp |
| Strand Bias (log2 ratio + to - strand density) | 0.2 |
| Multiplicity (# of sites on avg that occur together) | 1.00 |
| Motif File: | file (matrix) reverse opposite |

### Similar de novo motifs found

|  |  |  |  |  |  |  |  |
| --- | --- | --- | --- | --- | --- | --- | --- |
| Rank | Match Score | Redundant Motif | P-value | log P-value | % of Targets | % of Background | Motif file |
| 1 | 0.926 | C G T A A C T G C T A G G T A C C G T A A T C G A T C G A G T C A G T C A C G T A G T C A G T C | 1e-38 | -88.173550 | 2.19% | 0.19% | motif file (matrix) |
| 2 | 0.887 | C G T A A T C G A C T G A G T C C G T A A T C G A C T G A G T C A G T C A C G T A G T C | 1e-34 | -79.474166 | 2.09% | 0.20% | motif file (matrix) |
| 3 | 0.791 | T G C A T C G A A C T G A T G C C G T A C T A G A C T G T A G C A G T C A C G T | 1e-27 | -63.984601 | 2.55% | 0.46% | motif file (matrix) |
